# Three-Dimensional Imaging of Pulmonary Fibrotic Foci at the Alveolar Scale Using Tissue-Clearing Treatment with Staining Techniques of Extracellular Matrix

**DOI:** 10.1155/2020/8815231

**Published:** 2020-12-29

**Authors:** Kohei Togami, Hiroaki Ozaki, Yuki Yumita, Anri Kitayama, Hitoshi Tada, Sumio Chono

**Affiliations:** ^1^Department of Pharmaceutics, Faculty of Pharmaceutical Sciences, Hokkaido University of Science, 7-Jo 15-4-1 Maeda, Teine, Sapporo, Hokkaido 006-8585, Japan; ^2^Division of Pharmaceutics, Hokkaido Pharmaceutical University School of Pharmacy, 7-Jo 15-4-1 Maeda, Teine, Sapporo, Hokkaido 006-8585, Japan; ^3^Creation Research Institute of Life Science in KITA-no-DAICHI, 7-Jo 15-4-1 Maeda, Teine, Sapporo, Hokkaido 006-8585, Japan

## Abstract

Idiopathic pulmonary fibrosis is a progressive, chronic lung disease characterized by the accumulation of extracellular matrix proteins, including collagen and elastin. Imaging of extracellular matrix in fibrotic lungs is important for evaluating its pathological condition as well as the distribution of drugs to pulmonary focus sites and their therapeutic effects. In this study, we compared techniques of staining the extracellular matrix with optical tissue-clearing treatment for developing three-dimensional imaging methods for focus sites in pulmonary fibrosis. Mouse models of pulmonary fibrosis were prepared via the intrapulmonary administration of bleomycin. Fluorescent-labeled tomato lectin, collagen I antibody, and Col-F, which is a fluorescent probe for collagen and elastin, were used to compare the imaging of fibrotic foci in intact fibrotic lungs. These lung samples were cleared using the Clear^T2^ tissue-clearing technique. The cleared lungs were two dimensionally observed using laser-scanning confocal microscopy, and the images were compared with those of the lung tissue sections. Moreover, three-dimensional images were reconstructed from serial two-dimensional images. Fluorescent-labeled tomato lectin did not enable the visualization of fibrotic foci in cleared fibrotic lungs. Although collagen I in fibrotic lungs could be visualized via immunofluorescence staining, collagen I was clearly visible only until 40 *μ*m from the lung surface. Col-F staining facilitated the visualization of collagen and elastin to a depth of 120 *μ*m in cleared lung tissues. Furthermore, we visualized the three-dimensional extracellular matrix in cleared fibrotic lungs using Col-F, and the images provided better visualization than immunofluorescence staining. These results suggest that Clear^T2^ tissue-clearing treatment combined with Col-F staining represents a simple and rapid technique for imaging fibrotic foci in intact fibrotic lungs. This study provides important information for imaging various organs with extracellular matrix-related diseases.

## 1. Introduction

Idiopathic pulmonary fibrosis (IPF) is a progressive, chronic lung disease characterized by the accumulation of extracellular matrix proteins, including collagen and elastin, because of excessive synthesis by lung fibroblasts and myofibroblasts [1, 2]. This histological feature blocks the alveoli from opening on inspiration and shrinking on expiration, resulting in poor health-related quality of life with dyspnea and a low survival rate [3, 4]. Antifibrotic agents such as nintedanib and pirfenidone are used to treat IPF; however, these agents are inefficient and have various adverse effects. To develop a novel IPF therapy, it is necessary to accurately assess the severity of pulmonary fibrosis in both human patients and rodent experimental models. Three-dimensional (3D) imaging of the extracellular matrix, including collagen in isolated lung tissues of experimental animal models, would provide a valuable tool for evaluating the pathological condition as well as the distribution of drugs to pulmonary focus sites and their therapeutic effects.

Whole-body 3D imaging techniques, including magnetic resonance imaging (MRI), X-ray computed tomography, and positron emission tomography, have been used to analyze fibrotic lungs in several preclinical animal studies [5–7]. Although these imaging techniques enable the discrimination of pulmonary fibrosis, they have low resolution (millimeter level), and direct imaging of the elements such as collagen in pulmonary fibrotic foci is impossible. In contrast, micrographic assessment using tissue thin sections of stained fibrotic foci is limited to a thickness of several micrometers and 2D imaging. Because the alveolar structure is damaged by the processes of section preparation, tissue section imaging is neither technically complete nor accurate. Therefore, development of direct imaging techniques for visualizing extracellular matrix in fibrotic foci in intact lungs at high resolution is necessary.

We previously reported the usefulness of Clear^T2^ tissue-clearing treatment with fluorescent-labeled *Lycopersicon esculentum* lectin (tomato lectin) for the 3D imaging of intact healthy lungs at alveolar scale [8]. Because fluorescence-labeled tomato lectin stains many lung cells, including type I alveolar epithelial cells [9], the general alveolar structures covered by the alveolar epithelium of healthy lungs can be visualized. However, the alveolar epithelium is irreversible scarred in the first stage of IPF development [10]. Therefore, staining techniques that directly target fibrotic foci in cleared lung tissues are required. In this study, we described the 2D and 3D imaging techniques of fibrotic foci in intact fibrotic lungs using the Clear^T2^ tissue-clearing technique along with collagen staining methods, including immunostaining and Col-F (conjugate of physostigmine and fluorescein) as a low-molecular-weight fluorescent probe for collagen and elastin [11].

## 2. Materials and Methods

### 2.1. Materials and Animals

Bleomycin chlorate was purchased from Nippon Kayaku Company (Tokyo, Japan). Formamide was purchased from Fujifilm Wako Pure Chemicals Co., Ltd. (Osaka, Japan). Polyethylene glycol (8 kDa) was purchased from Sigma Aldrich Co. (St. Louis, MO, USA). DyLight 488-conjugated tomato lectin was purchased from Vector Laboratories Inc. (Burlingame, CA, USA). Col-F was purchased from ImmunoChemistry Technologies LLC (Bloomington, MN, USA). Male 5-week-old ICR mice weighing 26–28 g were purchased from Japan SLC (Shizuoka, Japan). The Laboratory Animal Center approved the animal experimental protocol (No. H29-001), which conformed to the Guiding Principles for the Care and Use of Experimental Animals at Hokkaido Pharmaceutical University.

### 2.2. Model Mouse of Bleomycin-Induced Pulmonary Fibrosis

Intrapulmonary administration of bleomycin chlorate (3 mg/kg) dissolved in phosphate-buffered saline solution (PBS, pH 7.4) was performed using Liquid MicroSprayers® (Model IA-1C; Penn-Century, Inc., Philadelphia, PA, USA) in mice under pentobarbital anesthesia. Fourteen days after administration, the mice were used as pulmonary fibrosis models in the following experiments. Pulmonary fibrosis was confirmed via Masson's trichrome staining of lung tissue sections and quantification of hydroxyproline in lung tissues, as previously reported [12, 13].

### 2.3. Frozen Lung Tissue Section

Cryobiopsy was performed under anesthesia via intraperitoneal administration of butorphanol tartrate (5 mg/kg) and sodium pentobarbital (50 mg/kg) as described previously [14]. Serial 8 *μ*m thick frozen sections were prepared using a cryostat and directly mounted onto glass slides (Matsunami Trading Company Ltd., Osaka, Japan).

### 2.4. Staining of Lung Tissue Sections

To evaluate the histological features of pulmonary fibrosis, lung tissue sections were stained using the Masson trichrome technique.

For the immunohistochemical staining of collagen I, the sections were washed twice in PBS for 5 min. After blocking with BLOXALL endogenous peroxidase, alkaline phosphatase blocking solution® (Vector Laboratories), and goat serum (Vector Laboratories), lung tissue sections were treated with a rabbit polyclonal anti-collagen I antibody (Abcam plc, Cambridge, UK; catalogue number: ab34710; 1 : 200 dilution) for 30 min at room temperature. The sections were treated with biotinylated anti-rabbit IgG secondary antibodies (Vector Laboratories; catalogue number: BA-1000; no dilution) for 30 min at room temperature. The sections were treated with avidin-biotin peroxidase complex (Vector Laboratories) for 30 min at room temperature. The staining was performed using 3,3′-diaminobenzidine tetrahydrochloride (Vector Laboratories), and the slides were counterstained using Mayer's hematoxylin. All sections were thoroughly washed with PBS for 5 min at room temperature between each staining step.

For staining with fluorescence-labeled tomato lectin, the lung sections were washed three times in PBS for 5 min and then treated with DyLight 488-conjugated tomato lectin (5 *μ*g/mL) for 1 h at room temperature. For staining with Col-F, the sections were washed three times in PBS for 5 min and then treated with Col-F (15 *μ*g/mL) for 1 h at room temperature.

### 2.5. Preparation of Mouse Lung Sample and Tissue-Clearing Treatment

**Figure 1:**
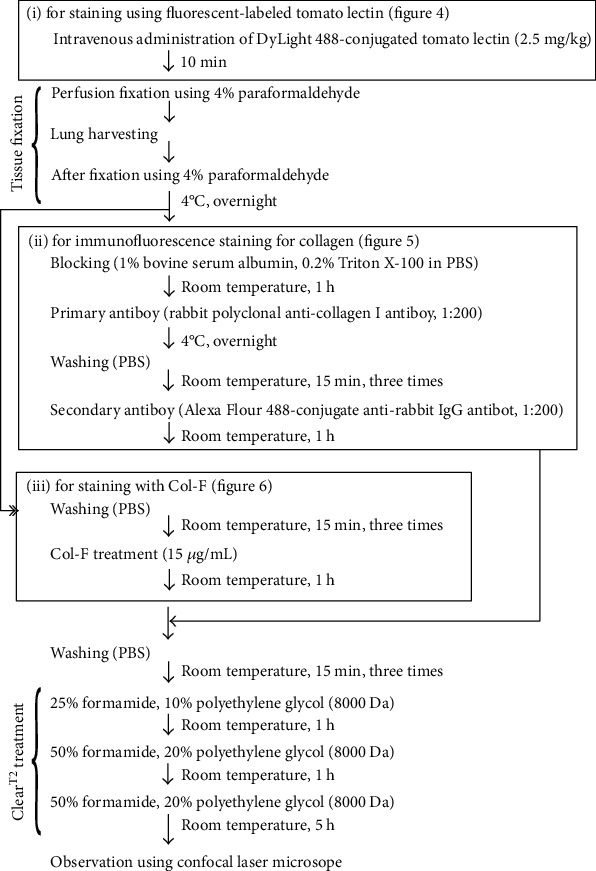
Experimental protocols for extracellular matrix staining and tissue-clearing treatment of fibrotic lungs in mice with bleomycin-induced pulmonary fibrosis.

Lung samples were stained with fluorescence-labeled tomato lectin, immunofluorescence staining, and Col-F and were cleared using the Clear^T2^ tissue-clearing technique protocols [15–17] (Figure 1). For the 3D imaging of fibrotic foci in the lungs, the cleared lungs were observed using laser-scanning confocal microscopy (LSM 700; Zeiss, Oberkochen, Germany) equipped with a Plan-Apochromat 10×/0.45M27 (Zeiss) objective lens (excitation: 488 nm laser; emission filter: 550 nm short-pass filter; dichroic beamsplitter: 493 nm).

### 2.6. Image Analysis

Images of Masson's trichrome staining of lung tissue sections and Col-F staining of cleared lung tissues were quantitatively analyzed using the ImageJ software version 1.53c (National Institutes of Health, Bethesda, MD, USA) [18]. In the images with Masson's trichrome staining, blue coloration indicated extracellular matrix localization and was separated and quantified as blue intensity per observation area. In the images of Col-F staining, fluorescence signals were quantified as intensity per observation area.

### 2.7. Statistical Analysis

Statistical analyses were performed using Student's *t*-test and the SPSS software version 27 (IBM Inc., Armonk, NY, USA). Differences were considered statistically significant at ^∗^*p* < 0.05.

## 3. Results

### 3.1. Accumulation of Collagen in Fibrotic Lungs

**Figure 2:**
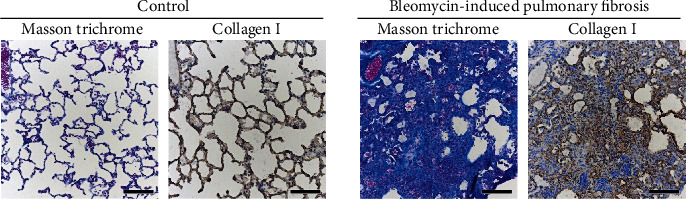
Pathologic features of the fibrotic lungs of mice with bleomycin-induced pulmonary fibrosis. After 14 days of bleomycin administration, frozen serial lung sections were prepared. Masson's trichrome staining and immunohistochemical staining were performed for the pathologic features and presence of collagen I, respectively. Scale bar is 100 *μ*m.

Masson's trichrome staining and collagen I immunostaining of frozen lung tissue sections of mice with bleomycin-induced pulmonary fibrosis are shown in Figure 2. The accumulation of collagen I in fibrotic foci on the alveoli was observed after 14 days of bleomycin administration.

### 3.2. Tissue-Clearing Ability of Fibrotic Lungs

**Figure 3:**
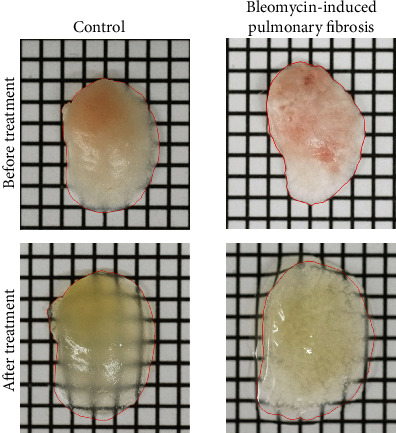
Transmission color images of fibrotic lung tissues from mice after Clear^T2^ tissue-clearing treatment. Each square represents 2 × 2 mm^2^. Red lines show the shape of lung lobe.

Transmitted images of fibrotic lungs after Clear^T2^ tissue-clearing treatment are shown in Figure 3. Grid lines could be observed under the cleared lungs of both control mice and mice with bleomycin-induced pulmonary fibrosis after Clear^T2^ treatment. As previously reported [15], Clear^T2^ tissue-clearing treatment increased the sizes of lung samples.

### 3.3. Staining with Fluorescence-Labeled Tomato Lectin

**Figure 4:**
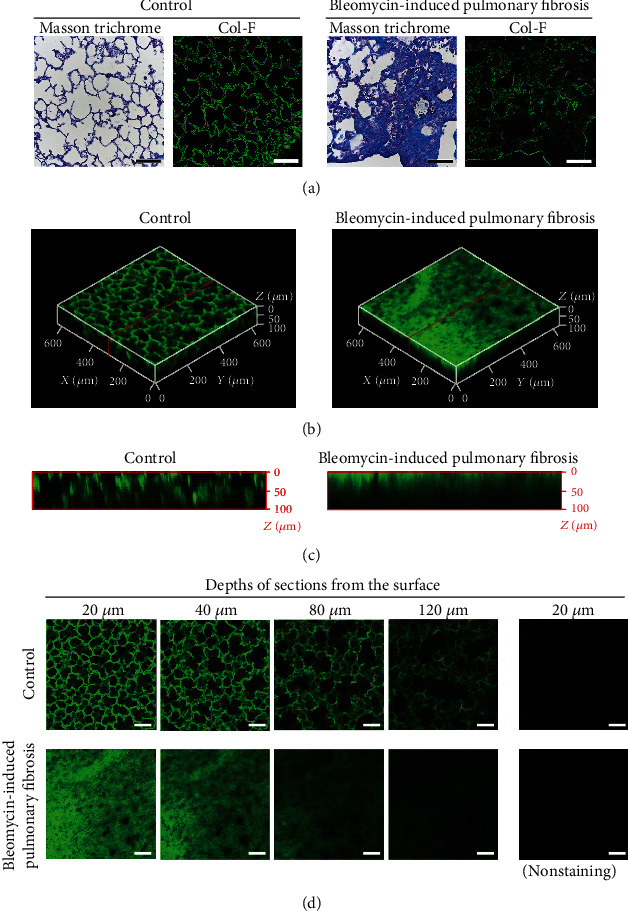
Fluorescence labeling of fibrotic lungs using fluorescence-conjugated tomato lectin. (a) Serial lung sections were stained using DyLight 488-conjugated tomato lectin and Masson's trichrome technique. (b–d) DyLight 488-conjugated tomato lectin (2.5 mg/kg) was intravenously administered to mice with bleomycin-induced pulmonary fibrosis. After Clear^T2^ tissue-clearing treatment, the lungs were observed using confocal microscopy. (b) Three-dimensional images were reconstructed from single-plane images. Each 5 *μ*m stack is 20–120 *μ*m below the lung surface. (c) The image of cross-sections indicated by the red lines in (b). (d) Single-plane images at the indicated depth (*μ*m) from the lung surface. Green fluorescence indicates the localization of tomato lectin. Scale bar is 100 *μ*m.

Tomato lectin-stained images of frozen lung tissue sections and intact cleared lung tissues are shown in Figure 4. In the lung sections, the fluorescence of tomato lectin was observed around the alveolar wall in control lungs but not on the fibrotic foci in fibrotic lungs (Figure 4(a)). In intact cleared lung tissues, the general alveolar structure of control lungs was observed in a 2D manner, which is similar to that observed in lung tissue sections (Figure 4(d)), and 3D visualization was possible (Figure 4(b)). In contrast, in the fibrotic lungs, the 2D and 3D images of intact cleared lung tissues differed (Figures 4(b) and 4(d)) from those of lung tissue sections (Figure 4(a)).

### 3.4. Immunostaining for Collagen I in Cleared Lungs

**Figure 5:**
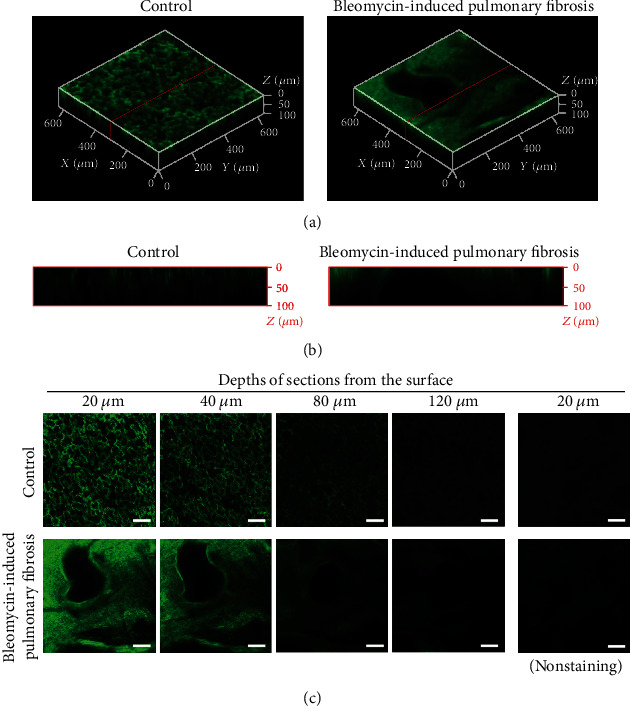
Three-dimensional images of collagen I stained using immunofluorescence in fibrotic lungs after Clear^T2^ tissue-clearing treatment. (a) Three-dimensional images reconstructed from single-plane images. Each 5 *μ*m stack is 20–120 *μ*m below the lung surface. (b) The image of cross-sections indicated by the red lines in (a). (c) Single-plane images at the indicated depth (*μ*m) from the lung surface. Green fluorescence indicates the localization of collagen I. The scale bar is 100 *μ*m.

Images of lungs subjected to immunofluorescence staining for collagen I with Clear^T2^ treatment are shown in Figure 5. Although 2D and 3D visualization of collagen I was achieved in both control and fibrotic cleared lungs, collagen I in these lungs was clearly visible only until a depth of only 40 *μ*m.

### 3.5. Col-F Staining for Extracellular Matrix in Cleared Lungs

**Figure 6:**
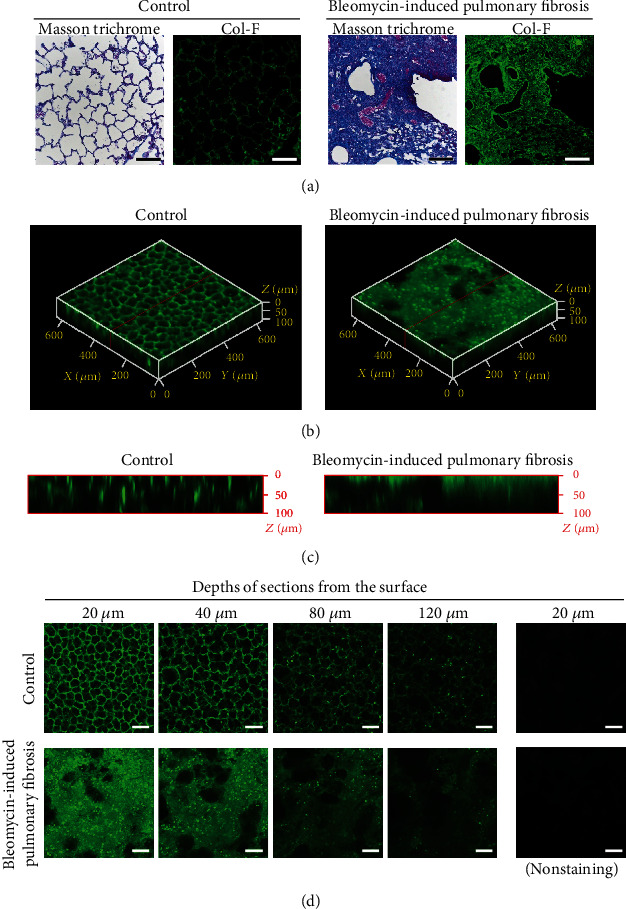
Fluorescence labeling of fibrotic foci in the lungs using Col-F. (a) Lung sections were stained using Col-F. Green fluorescence indicates the localization of Col-F. Scale bar is 100 *μ*m. (b–d) Fibrotic lungs were perfusion-fixed, excised, and stained using Col-F. After Clear^T2^ tissue-clearing treatment, the lungs were observed using confocal microscopy. (b) Three-dimensional images were reconstructed from single-plane images. Each 5 *μ*m stack is 20–120 *μ*m below the lung surface. (c) The image of cross-sections indicated by the red lines in (b). (d) Single-plane images at the indicated depth (*μ*m) from the lung surface. Green fluorescence indicates the localization of Col-F. The scale bar is 100 *μ*m.

**Figure 7:**
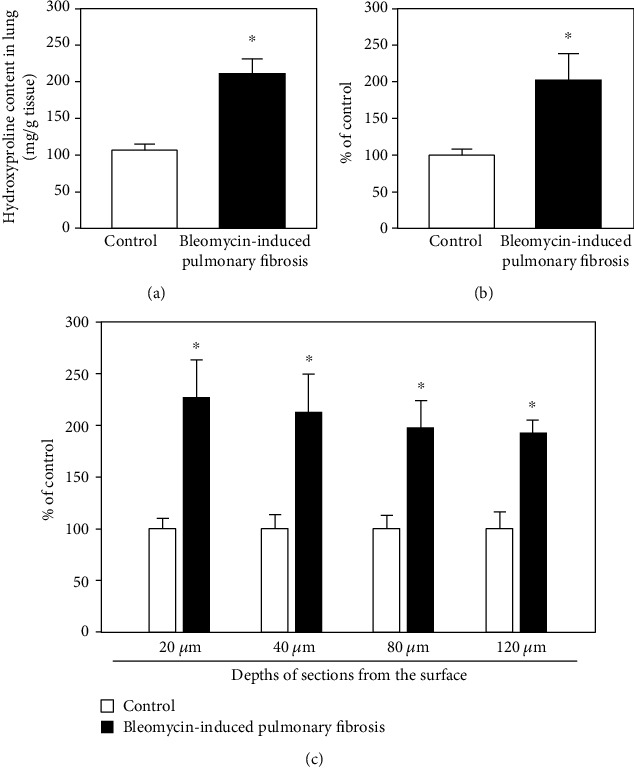
Quantitative analysis of fluorescence-labeled fibrotic foci in the lungs using Col-F after Clear^T2^ tissue-clearing treatment. (a) Hydroxyproline content in fibrotic mouse lungs. (b) Blue coloration indicating extracellular matrix localization on the lung tissue section. (c) Fluorescent intensities derived from single-plane images of Col-F at the indicated depth (*μ*m) from the lung surface. Each point represents the mean ± SD (*n* = 3). ^∗^*p* < 0.05.

Col-F–stained images of lung tissue sections and intact cleared lung tissues are shown in Figure 6. In the lung sections, Col-F fluorescence was observed in the fibrotic foci in fibrotic lungs (Figure 6(a)). In intact cleared lung tissues, the fluorescence of Col-F in fibrotic lungs was observed in a 2D manner, which is similar to that observed in lung sections (Figure 6(b)). Col-F–stained collagen and elastin were visible to a depth of 120 *μ*m in the cleared lung tissue (Figure 6(c)), representing threefold greater depth than that achieved with immunofluorescence staining (Figure 6(b)). The 3D visualization for collagen I was enabled in both control and fibrotic cleared lungs (Figure 6(d)), and the visualization was more clear than that achieved using immunofluorescence staining (Figure 5(c)). The fluorescence intensity of Col-F at each depth in the cleared fibrotic lung tissue was significantly higher (approximately two times) than that of control lungs (Figure 7). This relationship was similar to the hydroxyproline concentration in the lungs as an index of lung fibrosis and the result of the quantitative analysis of Masson's trichrome-stained images.

## 4. Discussion

In this study, we developed 2D and 3D imaging techniques to visualize the extracellular matrix deposited in pulmonary fibrotic foci using tissue-clearing treatment. Extracellular matrix accumulation in alveoli has also been observed in other lung diseases, including lung cancer and chronic obstructive pulmonary disease [19, 20]. Therefore, the Clear^T2^ technique might be useful for the clearing of lung tissues obtained from patients with these diseases. Several tissue-clearing techniques, such as CUBIC, CLARITY, and BABB, have been developed for lung tissues [21–23]. The Clear^T2^ technique has a moderate tissue-clearing ability with a short procedure time (7 h). Furthermore, this technique preserves the alveolar structure without causing delipidation because this technique does not require organic solvents and surface-active agents [24]. These advantages of tissue-clearing regents are believed to be useful for the visualization of drug distribution in the lungs [8].

Although we previously developed imaging techniques to visualize the general alveolar structure of healthy mice using the Clear^T2^ method and the intravenous administration of fluorescence-labeled tomato lectin [15], the fibrotic foci of mice with pulmonary fibrosis were not visualized using these techniques. Because tomato lectin binds to many epithelial and endothelial cell types, intravenous administration of tomato lectin is performed for vessel imaging [25]. However, tomato lectin is also used to evaluate vessel permeability via the detection of tomato lectin leakage from the vessel to the interstitial space [26]. Changes in the vessel permeability of the lungs are observed in patients with IPF and animals with bleomycin-induced pulmonary fibrosis [27, 28]. Thus, it is believed that tomato lectin penetrated and stained the lung interstitial space after intravenous administration in mice with bleomycin-induced pulmonary fibrosis. Therefore, the images of lung sections and cleared lungs after staining of tomato lectin are believed to differ. Tomato lectin does not bind to components of fibrotic foci, including lung fibroblasts and collagen. Predictably, tomato lectin is unsuitable for the imaging of fibrotic foci in tissues with pulmonary fibrosis.

In contrast to our findings, Decroix et al. succeeded in the deep 3D imaging of muscle tissues using the Clear^T2^ technique with immunostaining [16]. However, as immunostaining methods for Clear^T2^ treatment require Triton X-100, a surface-active agent, the release of alveolar surfactant elements in the lungs might be induced by the actions of this agent. Moreover, the use of surface-active agents for tissue-clearing treatment is unsuitable for several fluorescent lipophilic probes, such as DiI, which are used for blood vessel staining and labeling of drug carrier [8].

Several fluorescent probes for collagen staining have been previously reported, such as single-stranded collagen mimetic peptides [29], CNA35 labeled with fluorescent dye Oregon Green 488 [30], and collagen-hybridizing peptide [31]. However, these probes have mid-to-high molecular weights. Therefore, it may require longer treatment times and the addition of surface-active agents that cause major issues in observing cleared lung tissues, which may be necessary for fluorescent staining of intrapulmonary collagen. Col-F, a low-molecular-weight fluorescent probe, binds noncovalently to collagen and elastin [11]. Thus, it is believed that Col-F readily penetrated into fibrotic lung tissue and specifically stained collagen. Because this technique requires a shorter treatment time (<1 h) and no surface-active agents, the release of alveolar surfactant elements from the lung tissues is not believed to occur. Therefore, the technique might be useful to visualize not only fibrotic foci via Col-F staining but also as alveolar surfactant elements by other fluorescent labeling techniques and intrapulmonary distributed drugs after administration. In addition, because Col-F is used to stain sections of other tissues [11], Clear^T2^ treatment with Col-F staining might facilitate the 2D and 3D visualization of intact fibrotic liver and kidney tissues. Extracellular matrix production also occurs in and around tumor cells [20] and interrupts the delivery of antitumor agents [32]. Thus, this technique should be useful for visualizing the barrier composed of extracellular matrix around tumor cells.

Col-F was originally reported [11] as a probe with fluorescent-labeled collagen and elastin in freshly excised tissues. Col-F labels these extracellular matrix proteins only if cells in this tissue are live because live cells exclude Col-F. In this study, even in the lung tissue after fixation, the fluorescence image of Col-F showed a portion similar to the fibrotic foci observed on the Masson trichrome-stained images. This result indicates that the combination of Col-F with Clear^T2^ tissue-clearing treatment is useful to some extent for staining fibrous tissue. However, Col-F labels both collagen and elastin in the fibrotic lung. Moreover, the specific staining dots were observed in cleared lung tissues. Although the identity of these dots could not be determined, these stains may live tissues stained with Col-F in the original protocol used for the fixed lung tissue.

## 5. Conclusion

We developed a simple and rapid technique for the imaging of fibrotic foci in intact fibrotic lungs using Clear^T2^ tissue-clearing treatment with Col-F staining. This technique is useful for imaging collagen-related diseases in many organs.

## Data Availability

The data used to support the findings of this study are included in the article.

## References

[1] Maher T. M., Wells A. U., Laurent G. J. (2007). Idiopathic pulmonary fibrosis: multiple causes and multiple mechanisms?. *European Respiratory Journal*.

[2] Thannickal V. J., Henke C. A., Horowitz J. C. (2014). Matrix biology of idiopathic pulmonary fibrosis: a workshop report of the national heart, lung, and blood institute. *The American Journal of Pathology*.

[3] Belkin A., Swigris J. J. (2013). Health-related quality of life in idiopathic pulmonary fibrosis. *Current Opinion in Pulmonary Medicine*.

[4] Ley B., Collard H. R., King T. E. (2011). Clinical course and prediction of survival in idiopathic pulmonary fibrosis. *American Journal of Respiratory and Critical Care Medicine*.

[5] Désogère P., Tapias L. F., Rietz T. A. (2017). Optimization of a collagen-targeted pet probe for molecular imaging of pulmonary fibrosis. *Journal of Nuclear Medicine*.

[6] Scotton C. J., Hayes B., Alexander R. (2013). Ex vivomicro-computed tomography analysis of bleomycin-induced lung fibrosis for preclinical drug evaluation. *European Respiratory Journal*.

[7] Velde G. V., de Langhe E., Poelmans J., Dresselaers T., Lories R. J., Himmelreich U. (2014). Magnetic resonance imaging for noninvasive assessment of lung fibrosis onset and Progression. *Investigative Radiology*.

[8] Togami K., Daisho T., Yumita Y., Kitayama A., Tada H., Chono S. (2019). Evaluation of various tissue-clearing techniques for the three-dimensional visualization of liposome distribution in mouse lungs at the alveolar scale. *International Journal of Pharmaceutics*.

[9] Kasper M., Singh G. (1995). Epithelial lung cell marker: current tools for cell typing. *Histology and Histopathology*.

[10] Plantier L., Cazes A., Dinh-Xuan A. T., Bancal C., Marchand-Adam S., Crestani B. (2018). Physiology of the lung in idiopathic pulmonary fibrosis. *European Respiratory Society*.

[11] Biela E., Galas J., Lee B., Johnson G. L., Darzynkiewicz Z., Dobrucki J. W. (2013). Col-F, a fluorescent probe for ex vivo confocal imaging of collagen and elastin in animal tissues. *Cytometry Part A*.

[12] Izbicki G., Segel M. J., Christensen T. G., Conner M. W., Breuer R. (2002). Time course of bleomycin-induced lung fibrosis. *International Journal of Experimental Pathology*.

[13] Hutson P. R., Crawford M. E., Sorkness R. L. (2003). Liquid chromatographic determination of hydroxyproline in tissue samples. *Journal of Chromatography B*.

[14] Togami K., Chono S., Tada H. (2016). Alteration in intrapulmonary pharmacokinetics of aerosolized model compounds due to disruption of the alveolar epithelial barriers following bleomycin- induced pulmonary fibrosis in rats. *Journal of Pharmaceutical Sciences*.

[15] Togami K., Kitayama A., Daisho T., Wang R., Tada H., Chono S. (2018). Tissue-clearing techniques enable three-dimensional visualization of aerosolized model compound and lung structure at the alveolar scale. *Biological & Pharmaceutical Bulletin*.

[16] Decroix L., Van Muylder V., Desender L., Sampaolesi M., Thorrez L. (2015). Tissue clearing for confocal imaging of native and bio-artificial skeletal muscle. *Biotechnic & Histochemistry*.

[17] Kuwajima T., Sitko A. A., Bhansali P., Jurgens C., Guido W., Mason C. (2013). ClearT: a detergent- and solvent-free clearing method for neuronal and non-neuronal tissue. *Development*.

[18] Schneider C. A., Rasband W. S., Eliceiri K. W. (2012). NIH Image to ImageJ: 25 years of image analysis. *Nature Methods*.

[19] Bihlet A. R., Karsdal M. A., Sand J. M. (2017). Biomarkers of extracellular matrix turnover are associated with emphysema and eosinophilic-bronchitis in COPD. *Respiratory Research*.

[20] Fang S., Dai Y., Mei Y. (2019). Clinical significance and biological role of cancer-derived type I collagen in lung and esophageal cancers. *Thoracic Cancer*.

[21] Lee H., Park J. H., Seo I., Park S. H., Kim S. (2014). Improved application of the electrophoretic tissue clearing technology, CLARITY, to intact solid organs including brain, pancreas, liver, kidney, lung, and intestine. *BMC Developmental Biology*.

[22] Nojima S., Susaki E. A., Yoshida K. (2017). CUBIC pathology: three-dimensional imaging for pathological diagnosis. *Scientific Reports*.

[23] Scott G. D., Blum E. D., Fryer A. D., Jacoby D. B. (2014). Tissue optical clearing, three-dimensional imaging, and computer morphometry in whole mouse lungs and human airways. *American Journal of Respiratory Cell and Molecular Biology*.

[24] Seo J., Choe M., Kim S. Y. (2016). Clearing and labeling techniques for large-scale biological tissues. *Molecular Cell*.

[25] Robertson R. T., Levine S. T., Haynes S. M. (2015). Use of labeled tomato lectin for imaging vasculature structures. *Histochemistry and Cell Biology*.

[26] Morikawa S., Baluk P., Kaidoh T., Haskell A., Jain R. K., McDonald D. M. (2002). Abnormalities in pericytes on blood vessels and endothelial sprouts in tumors. *The American Journal of Pathology*.

[27] McKeown S., Richter A. G., O'Kane C., McAuley D. F., Thickett D. R. (2009). MMP expression and abnormal lung permeability are important determinants of outcome in IPF. *European Respiratory Journal*.

[28] Yin Q., Nan H., Yan L. (2012). Alteration of tight junctions in pulmonary microvascular endothelial cells in bleomycin-treated rats. *Experimental and Toxicologic Pathology*.

[29] Li Y., Ho D., Meng H. (2012). Direct detection of collagenous proteins by fluorescently labeled collagen mimetic peptides. *Bioconjugate Chemistry*.

[30] Aper S. J. A., van Spreeuwel A. C. C., van Turnhout M. C. (2014). Colorful protein-based fluorescent probes for collagen imaging. *PLoS One*.

[31] Hwang J., Huang Y., Burwell T. J. (2017). In situ imaging of tissue remodeling with collagen hybridizing peptides. *ACS Nano*.

[32] Weniger M., Honselmann K. C., Liss A. (2018). The extracellular matrix and pancreatic cancer: a complex relationship. *Cancers*.

